# Upper and Lower Leaf Side Detection with Machine Learning Methods

**DOI:** 10.3390/s22072696

**Published:** 2022-03-31

**Authors:** Rodica Gabriela Dawod, Ciprian Dobre

**Affiliations:** 1 Faculty of Automatic Control and Computer Science, University Politehnica of Bucharest, 060042 Bucharest, Romania; ciprian.dobre@upb.ro; 2 National Institute for Research and Development in Informatics, 011455 Bucharest, Romania

**Keywords:** foliar disease identification, leaf vein segmentation, leaf side detection, convolutional neural network

## Abstract

Recent studies have approached the identification of foliar plant diseases using artificial intelligence, but in these works, classification is achieved using only one side of the leaf. Phytopathology specifies that there are diseases that show similar symptoms on the upper part of the leaf, but different ones on the lower side. An improvement in accuracy can be achieved if the symptoms of both sides of the leaf are considered when classifying plant diseases. In this context, it is necessary to establish whether the captured image represents the leaf on its upper or lower side. From the research conducted using botany books, we can conclude that a useful classification feature is color, because the sun-facing part is greener, while the opposite side is shaded. A second feature is the thickness of the primary and secondary veins. The veins of a leaf are more prominent on the lower side, compared to the upper side. A third feature corresponds to the concave shape of the leaf on its upper part and its convex shape on the lower part. In this study, we aim to achieve upper and lower leaf side classification using both deep learning methods and machine learning models.

## 1. Introduction

Increasing productivity in agriculture is an objective addressed worldwide, along with protecting the environment and reducing pollution. Farmers must face the threats of climate change, as well as the appearance of diseases and pests. Excessive amounts of rain, drought or even the absence of snow contribute equally to the partial loss of harvests [1]. Precision agriculture is one of the tools of the future, which helps farmers through digital solutions, created with the help of artificial intelligence.

In this context, there has been an increase in research efforts concerning the digital solutions used for the detection of plant diseases. Increasing coverage with communication networks in rural areas, along with the digital solutions offered to farmers, can contribute to the early detection of diseases, which allows for timely plant protection measures to be taken [2]. Plant disease detection studies can be conducted using images captured by satellites, drones or mobile phones. Through satellite images, multiple vegetation indexes can be analyzed, allowing farmers to monitor their crops. The most common vegetation indexes are the Normalized Difference Vegetation Index (NDVI), the Green Normalized Difference Vegetation Index (GNDVI) and the Enhanced Vegetation Index (EVI).

While studies based on images taken by drones or satellites are focused on detecting regions of the field that are affected by diseases or recommending agricultural operations, studies based on images taken by cameras or mobile phones focus on the classification of the disease, because these images have the necessary resolution for this type of study.

Based on the need to classify a disease accurately, the broad framework of this research aims to improve the identification results by studying the possibility of considering the symptoms of both sides of the leaf in disease classification. The present work represents a first chapter of the research, namely, the classification of an image of a leaf in the upper or lower part.

Studies in the field of disease detection with machine learning algorithms are based on image processing and the segmentation of lesions, followed by the extraction of features to be used for classification. By using different machine learning algorithms, researchers hope to achieve a higher classification accuracy. J. Liu et al. [3] used the Morphological Weight Adaptive image denoising method followed by the K-Means ++ clustering algorithm and the Watershed algorithm to segment an image showing sunflower leaf disease. Color and texture features were extracted from the diseased areas and the Random Forest algorithm was used for classification. S. Vijay [4] used the Particle Swarm Optimization algorithm for the segmentation of sunflower leaf images. A. Akhtar et al. [5] used Otsu’s algorithm for segmentation. The extraction of features was performed using three techniques: Statistical Features, Discrete Cosine Transform and Discrete Wavelet Transform. The classification was performed with K-Nearest Neighbor, Support Vector Machine, Decision Tree, Naïve Bayesian and Recurrent Neural Network. J.G.A. Barbedo et al. [6] proposed a method of disease classification based on color transformations, color histograms and a pairwise-based classification system. His study used a dataset of 12 plants with a total of 82 disorders The number of images used on the type of plant and disease varied between 2 and 76. The overall accuracy of the algorithm was 58% and the individual accuracy per plant type varied between 40% and 76%.

In the case of machine learning algorithms, the number of images in the dataset does not need to be large. In the papers presented above, the dataset is either composed of images taken in laboratory conditions or the images taken from the field are processed manually or automatically to remove the background elements and focus on leaves and diseased areas. Given the small number of images used in the analyzed studies, the accuracy of the classification is low when testing field images, even though the works indicate a high accuracy.

A more recent and modern classification approach is based on deep learning algorithms. This type of model does not need explicit features, but they are obtained based on optimization methods.

The variation in the studies is caused by the dataset used (containing field images or laboratory-taken images), the realization of the classification using images of the entire leaf or only the areas with lesions, the use of different preprocessing techniques on input images, as well as the use of several types of convolutional neural networks. In the case of deep learning algorithms, a high number of images is needed to train a network. Many research articles have been based on the Plant Village [7] dataset. Images from this database are laboratory images, containing one leaf on a uniform background and without annotations related to the upper or lower leaf side. The performance results of detection algorithms are good for images of the same nature, but a high accuracy decrease is observed when classifying field images.

P. Sharma et al. [8] used a dataset combining images from Plant Village and Internet sources. A convolutional neural network developed in a previous work was used to classify two types of images, the first containing the entire leaf, and the second only images with lesions. A higher classification accuracy was obtained in the case of images containing lesions. S.P. Mohanty et al. [9] also performed classification using Plant Village with full and segmented lesions, and, for training, they selected AlexNet and GoogLeNet, two popular CNN models. S. Sladojevic et al. [10] used CafeNet as a classification network and a set of images from the Internet. E. C. Too et al. [11] used Plant Village as a dataset for classification and several CNN models, including VGG 16, Inception V4, and ResNet.

Garg et al. [12] used Mask R-CNN to segment the leaves, followed by segmentation of the diseased region using the fully convolutional network (FCN). The dataset was composed of images of maize leaves with Northern Leaf Blight (NLB) disease. Ke Lin et al. [13] used in her study the U-Net model, trained to detect Powdery Mildew disease in cucumber leaves. Fifty infected leaves were collected from fields and were photographed in a controlled environment, placing the leaf on a black background. Images were manually annotated and by using augmentation, the number of leaves and their annotations were increased to 10,000 instances.

The works mentioned above do not address the problem of classification using both sides of the leaf. The used images do not contain the diversity of background elements from nature and the algorithms used in segmentation are specific to the set of images.

Phytopathology books and previous research carried out with the help of artificial intelligence show that there are foliar diseases with similar manifestation, a case in which it is difficult to achieve automatic detection with a high degree of accuracy. For some of these diseases, the analysis of the lower part of the leaf provides additional information, which can be used to improve the results obtained in classification.

A favoring factor in the appearance of diseases is represented by the microclimate created by large leaves after it rains. The large leaves will create an area with a different environment than the outer one, mainly wetter and cooler, very conducive to diseases of the plasmopara group, such as Downy Mildew. When the sun comes out again, the upper side is warm and dry while the lower side is moist and dark.

In the case of the sunflower, Alternaria and Septoria are examples of diseases that show similar symptoms on the top of the leaf but differ in the lower part. White Rust and Downy Mildew are two diseases that can also be confused.

With the ultimate goal of improving the algorithms for classifying diseases in plants, this paper studies the possibility of classifying the leaf in the upper–lower parts.

The used dataset contained only images of sunflower leaves; a test on a diverse set of plants will be conducted in another study. The dataset contained sunflower images taken from the field and pre-processed by manual cropping and resizing to focus on leaf and veins.

Both deep learning and machine learning models were used for classification.

A convolutional neural network (CNN) was considered for classification due to the performances obtained in previous studies. A network of the ResNet type [14] was used with two architectures, ResNet50 and ResNet152. This type of network, created in 2015 by researchers at Microsoft Research, was chosen due to the reduced time required for training, the low storage space of the learned parameters and its high accuracy.

For machine learning models, the first step was to identify relevant characteristics.

Because the thickness of the veins is more prominent on the lower part of the leaf compared to the upper one, a first attempt to extract features was conducted using images obtained from edge segmentation algorithms. Edge segmentation methods can be grouped into two categories, depending on the order of the derivative. First order derivative algorithms are called search based, while second order derivative algorithms are called zero-crossing. The segmentation techniques used in this study are Canny Edge Detection, introduced in 1986 by J. Canny [15]; Prewitt Edge Detection developed by J.M.S. Prewitt in 1970 [16]; Sobel operator first presented in 1968 by I. Sobel [17]; and Roberts cross operator proposed by L. Roberts in 1963.

Along with the thickness of the veins, the color, shape and texture of the leaf can play a role in the classification.

The machine learning classification was achieved with five models: Support Vector Machine (SVM), introduced in 1995 by C. Cortes [18]; Decision Tree (DT), first discussed in 1984 [19]; Random Forest (RF), created in 1995 by Tin Kam Ho [20]; the K-Nearest Neighbor (KNN) method, developed by Evelyn Fix and Joseph Hodges in 1951 [21]; and Gaussian Naive Bayes (GaussianNB), based on the Bayes Theorem [22].

In this paper, we present one of the first studies designed to shed light on the influence of the upper–lower side of a leaf in image-based diagnosis of plant diseases. In agronomy it is well known that many plant diseases present different symptoms depending on the side of the leaf. To us, it is somewhat surprising that this feature is not more widely used in AI and machine learning algorithms designed for image-based plant disease detection, as our investigation of state-of-the-art shows.

This study aims to apply various methods to classify an image containing a leaf in the upper–lower side. We start from the use of classical methods of segmentation of the edges to analyze whether the thickness of the primary or secondary veins can be used as a feature in the classification. We continue with the testing of the classification using the ResNet convolutional network in 2 architectures, with 50 and 152 levels, and the set of unprocessed images. Finally, we test the classification with five machine learning models, including Support Vector Machine (SVM), Decision Tree (DT), Random Forest (RF), K-Nearest Neighbor (KNN) and Gaussian Naïve Bayes (GaussianNB), using color, texture and shape as features.

To the best of our knowledge, the classification of diseases based on the symptoms that are present on both sides of the leaves has not been carried out before. The classification in the upper–lower leaf side is a prerequisite of the extended study.

Based on the obtained results, we can decide whether the introduction of both parts of the leaf in the classification of diseases is a viable objective, which can bring real improvements to the solutions currently used. The rest of the paper is organized as follows: Section 2 presents the methodology; Section 3 presents the achieved results; Section 4 contains the discussion; and, finally, Section 5 contains our conclusions.

## 2. Materials and Methods

### 2.1. Anatomy of a Leaf

Depending on their position in the limb and thickness, veins are classified as main, secondary, or tertiary. The veins of the leaf are more prominent on the lower side compared to the upper side. The side oriented towards the sun is greener and has a shiny protective layer, preventing loss of water due to evaporation. The opposite side faces the shadow. Another characteristic is that the upper side of a leaf has a concave form, while the underside is convex.

**Figure 1:**
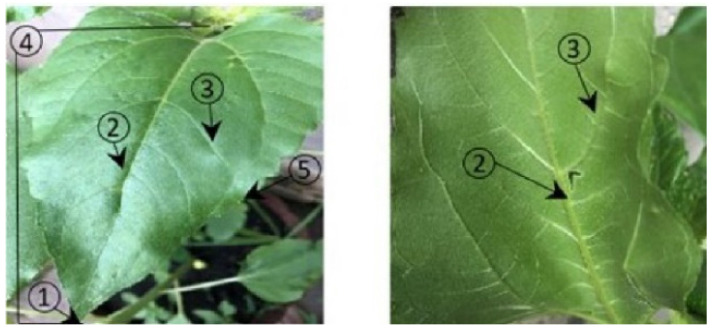
Leaf parts on the upper and lower side. (**1**) apex; (**2**) midvein (primary vein); (**3**) secondary vein; (**4**) lamina; (**5**) leaf margin.

According to the anatomy of a sunflower leaf, identifying which side of the leaf is the top versus underside is not always easy, but the criteria listed above can contribute to a correct classification. Figure 1 shows the components of the leaf.

### 2.2. Sunflower Diseases and Symptoms

From the disease symptoms extracted from journals or phytopathology books [23,24] and presented in Table 1, we can observe that disease manifestation on the upper side of leaf is different than on the lower side. Being able to identify the leaf side would lead to improved classification results, especially when symptoms are similar on one side of leaf, but different on the other side.

According to the literature, the presence of pycnidia on the lower side of the leaf is the best means of distinguishing leaf spots caused by Septoria from those caused by Alternaria. White rust presents structures similar to downy mildew.

### 2.3. Dataset

In general, the images used by previous studies were taken in laboratory conditions, but we are interested in how to implement an analysis and diagnosis system in real conditions. The images used in this study were taken from the field, using an iPhone equipped with a high-performance camera. The dataset contained both images of the upper and lower parts of healthy leaves, as well as images of diseased leaves.

**Figure 2:**
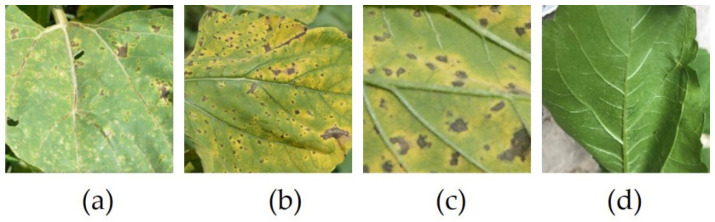
Images from the dataset. (**a**) Upper; (**b**) Upper; (**c**) Lower; (**d**) Lower.

The purpose of the study was to classify the leaf in the upper/lower part; to achieve this goal, disruptive elements, such as background elements and the human hand holding the leaf to be able to photograph the lower face, were removed. Images were manually processed by cropping to focus on the leaf and resized to 224 × 224. Table 2 presents the structure of the dataset and Figure 2 shows sample images from the dataset.

### 2.4. Extraction of Leaf Characteristics

#### 2.4.1. Edge Detection

In this section, we present the four edge detection methods used in this study. The segmentation was carried out in order to observe whether the thickness of the veins can be used to classify the leaf surface. These algorithms were selected due to the performance results obtained over time in image processing. In edge detection, image smoothing is a required pre-processing step and Gaussian smoothing is the most typical method applied.

Canny Edge Detector is an edge operator able to detect a wide range of edges in the image. Developed in 1986 by J.F. Canny, the algorithm is based on the first derivative of a Gaussian function. The operator has a multistage algorithm, starting with image noise removal achieved with a Gaussian filter. After finding the intensity gradients, thresholding is applied to determine potential edges. Although the appearance of this algorithm took place over thirty years ago, the Canny Edge Detector is still a state-of-the-art technique in this area.

Prewitt Edge Detection is an edge detection operator, introduced by J.M.S. Prewitt, based on the calculation of the gradient of the image intensity. At each point, we obtain the direction of the largest intensity increase and the value of change. The operation is based on convolving a small filter on the vertical and horizontal direction of the image and it is not expensive in terms of computation. The kernels used for convolution have a size of 3 × 3 and fixed coefficients.

Sobel Operator is similar to Prewitt. It is also based on the first derivative, and it is also detecting edges in both the vertical and horizontal directions. The main difference is given by the fact that the coefficients of convolution kernels can be adjusted, as long as they respect the property of derivative masks. The Sobel operator will find more edges than the Prewitt operator, because more weight was given to the pixel intensities around the edges.

The Roberts Operator was proposed by Lawrence Roberts in 1963 and it is one of the first edge detectors. Its functionality is based on convolution kernels, with a size of 2 × 2. Since the kernel is small, one of the disadvantages of this algorithm is the sensitivity to noise. The Sobel Operator performs better in this respect. The speed at which the calculation is performed is one of the advantages of using this operator.

#### 2.4.2. Feature Extraction

For the classification methods based on machine learning algorithms, starting from the given set of images, it is necessary to reduce the dimension and extract information, necessary for classification. The relevant features for the upper–lower leaf side classification are colors, textures, and shapes. Color is one of the most commonly used features in the classification of images, since, in addition to stability, it is also invariant to rotation or image scaling.

Color features are extracted either using the color histogram or the color moments. Color moments are measures that describe the color distribution in an image and allow us to compare how similar two images are, depending on the color. The literature recommends using only the first three color moments as features: mean, standard deviation and skewness. These three-color moments are computed per channel.

To describe the texture of an image, the concept of Haralick features is widely used. Those features are computed over the entire image. In [25], Haralick proposed 13 values extracted from the Gray Level Co-Occurrence Matrix (GLCM) and used to quantify texture. In disease classification, Haralick features could be useful not only in disease classification, but also in our attempt to identify if a given image represents the upper side of a leaf or the underside.

One of the classic methods used to extract the shape of an object from an image is based on a paper published in 1962 by Ming-Kuei Hu [26], calculating 7 moments that are invariant to translation, scale, and rotation. In order to obtain a good shape characterization of an object, we should either use a segmented binary image or the boundary of an object. The segmented image can be obtained via thresholding, or in the case of boundary, we can use edge detection.

When an image contains multiple objects, calculating Hu moments become irrelevant, since the calculation starts from the center of the object. Given this information, using Hu moments for detecting the shape of the lesions caused by diseases becomes more complex, since we must first identify each lesion from a leaf.

### 2.5. Classification Methods

#### 2.5.1. Convolutional Neural Network

CNN are state-of-the-art models used for image classification. Their function is based on the convolution operation. During the training phase, the network learns the necessary parameters for classification: weights and bias. Training can start from scratch, or transfer learning can be applied. Initial training is usually achieved using datasets, such as ImageNet or Cifar10. In transfer learning, pre-trained weights are loaded into the network and then refined during training on the new dataset.

The ResNet model used in this study won the first place on the ILSVRC 2015 contest and proposes a new architecture called Residual Network. In addition to a high classification accuracy, this network is fast to train and uses a small storage space for the learned parameters. The RestNet model was pre-trained on the ImageNet [27] dataset and then trained on the new dataset.

#### 2.5.2. Machine Learning Models

In this section, we present the five machine learning models used in this study. These methods were selected because they offered robust results over time in multiple areas of research.

The Support Vector Machine (SVM) is a supervised machine learning model, developed at AT&T Bell Laboratories, used for classification and regression analysis. It is a kernel-based algorithm with applicability in multiple fields, from character recognition and the classification of satellite images to biology and other sciences. It was built to classify two-class tasks and extended to multiclass SVM. The multiclass classification is resolved by reducing the multiclass problem to a set of multiple binary classification tasks.

Decision Trees (DT) are very popular supervised learning algorithms due to their simplicity. The algorithm to build the tree is usually a top-down approach, taking, on each step, the path that splits the given data the best. Building the decision tree is based on a greedy algorithm. Decision Trees are used for both classification and regression tasks. This is an algorithm that is easy to understand, as it can use both numerical and categorical data and performs well on large datasets. Although the algorithm has many advantages, one of the observed disadvantages is the fact that a small change in the training set can lead to a radical change of the tree and, consequently, to a change in the result of the classification.

Random Forests (RF) are used for both regression and classification and outperform decision trees. The problem of overfitting to the training set encountered in Decision Trees is corrected in this new method. RFs are an ensemble learning method, constructing multiple decision trees during the training time. The class selected by the majority of trees is the output of the random forest in the case of classification. Random Forests are less interpretable than decision trees.

K-Nearest Neighbor (KNN) is a non-parametric classification method used for classification and regression. The class of a data point is determined by the majority voting principle. The classes of the k-closest points are checked, and prediction is given by majority. In the case of regression, the result is the mean value of the k-closest points. To measure the distance between two points in a two-dimensional space, the Euclidean distance is usually used, but the concept becomes more complex if referring to text classification or other areas. This algorithm presents an issue when the class distribution is imbalanced since the frequent class dominates the prediction. The choice of k also has a major influence on the outcome of the classification. For k = 1, the model is too specific, while for a high k, the model could be too general. The selection of k is currently achieved with automatic methods.

Gaussian Naive Bayes (GaussianNB) is a probabilistic machine learning model used for classification and based on the Bayes theorem. The assumption of the algorithm is that features are independent of each other. Even if this naive assumption is not true in most cases, the algorithm performs better than expected. Gaussian Naïve Bayes is part of a collection of classification algorithms called Naive Bayes classifiers and the assumption is that values assigned to each feature are distributed according to a Gaussian distribution.

## 3. Results

### 3.1. Edge Detection for the Segmentation of Veins

**Figure 3:**
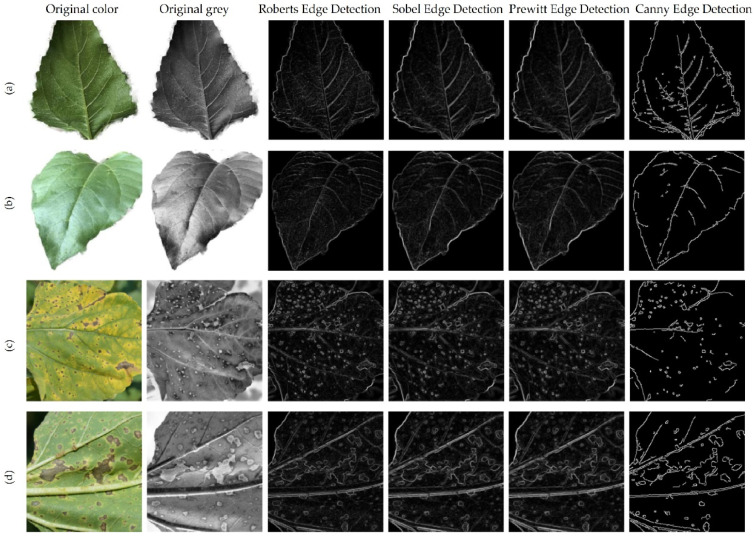
Edge detection methods applied to healthy and diseased leaves. (**a**) Lower side of healthy leaf; (**b**) Upper side of healthy leaf; (**c**) Upper side of diseased leaf; (**d**) Lower side of diseased leaf.

In order to segment leaf veins in the image, several edge detection methods were tested, such as Canny, Sobel, Roberts, and Prewitt. Edge detection was tested both on healthy and diseased leaves and the results are presented in Figure 3.

In Figure 3a,b, which show healthy leaves, the main and secondary veins could be relatively well segmented, but in Figure 3c,d, which contain diseased areas, we obtained a mix of veins and diseased areas from edge segmentation. The results are even more complex in images with a background that contains many details. Testing edge segmentation with multiple images did not suggest a possible classification method based on segmented images. The study continued with the attempt to classify the upper versus the lower part of the leaf, using a CNN network receiving images focused on the veins as training data.

### 3.2. Classification with CNN Models

To test the possibility of classifying the upper–lower leaf side, a dataset was created, which is presented in Table 2. From the original set of images collected in the field, we extracted images relevant for the two groups of classification. Cropping was applied as pre-processing to remove unwanted areas from the images. Images were resized to 224 × 224 after cropping.

According to the anatomy of a sunflower leaf, identifying which side of the leaf is the top and which is the underside is not always an easy task. The side oriented towards the sun is greener and has a shiny protective layer, preventing the loss of water due to evaporation. The opposite side faces the shadow.

The veins of the leaf are more prominent in the lower side, compared to the upper side. This is a feature of cordate leaves, but it might not be applicable to other types of leaves, such as corn leaves.

Another characteristic is that the upper side of the leaf has a concave form, while the underside is convex.

**Figure 4:**
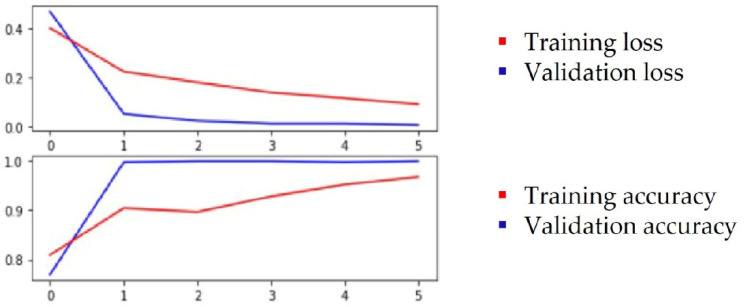
Plot of the training and validation loss and accuracy for ResNet152.

**Figure 5:**
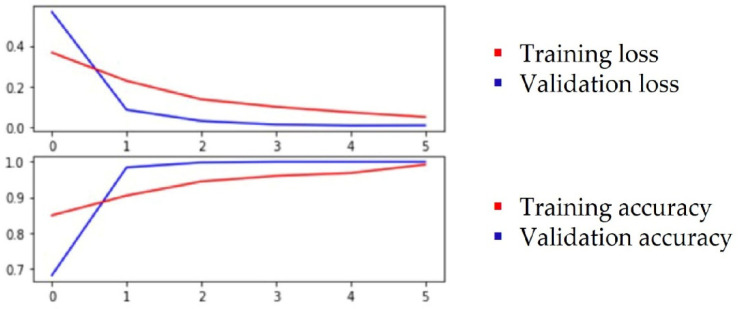
Plot of the training and validation loss and accuracy for ResNet50.

Based on the above observations, we assumed that, for classification, relevant features are associated to leaf color, the thickness of the veins and the concave/convex orientation of the leaf. Through the feature visualization of convolutional neural networks, past studies learned that, in lower levels, networks will learn simple edge and texture detectors, while in higher layers, they learn more abstract parts and object detectors. Both the ResNet50 and ResNet152 networks were selected for this test and the test was performed with color images. Transfer learning was applied with pretrained weights on an ImageNet dataset. Different hyperparameters were used for training and Table 3 shows the best metrics that were obtained when the training was conducted with cross-entropy loss, Stochastic Gradient Descent (SDG) optimizer, 0.001 learning rate, batch size of 42 and 6 epochs. The evolution of training results for ResNet152 is plotted in Figure 4 and for ResNet50 in Figure 5.

The models were adjusted with the addition of a Global Average Pooling layer followed by a dense layer, using SoftMax for activation and two neurons representing the two classes.

**Figure 6:**
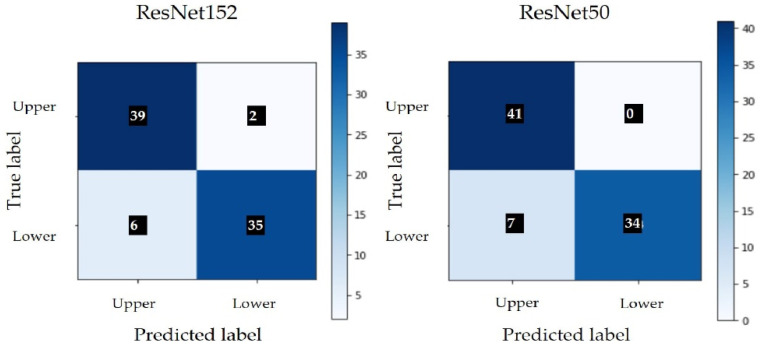
Confusion matrix without normalization.

The classification results are presented in Table 4 and Figure 6 shows the confusion matrix obtained in both architectures. Class 0 represents the upper side and class 1 represents the underside of the leaf.

The results obtained by applying the CNN model seem promising, but, as the number of images used is relatively small, the testing of the model should continue with an extended dataset. Both models had a similar performance and showed a tendency to misclassify images of the lower leaf side.

**Figure 7:**
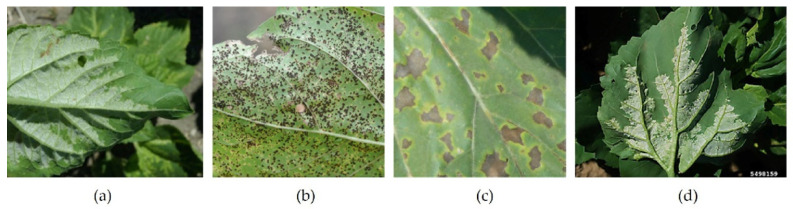
Images misclassified by the ResNet50 network: (**a**) Downy Mildew; (**b**) Rust; (**c**) Alternaria; (**d**) Downy Mildew.

The images used in this test were pre-processed manually by using cropping to extract a region of interest that contained the leaf and veins. When testing with images were taken in the field, without pre-processing, the expectation was that performance would drop, unless a vein segmentation processing system was implemented, which is possible with help of Mark R-CNN. Figure 7 shows misclassified images. Of the seven images with a wrong classification in ResNet50, three were not included in Figure 7, as they are similar to images Figure 7a,d [28] modified by rotation. These images are different from the set used for training because they show background elements.

A reason for the misclassification of Figure 7a,d may be the presence of background elements. We also noticed that those images showed an advanced degree of manifestation of the disease, which can influence the result. If Figure 7a,b clearly represent a missed classification, Figure 7c could be an upper side, wrongly considered as the underside of a leaf. Even visually, Figure 7c cannot be classified with 100% accuracy.

### 3.3. Classification with Machine Learning Models

The experiments in this section were conducted to compare the results obtained in the classification of disease using a CNN network with the results obtained using machine learning models.

Starting from the same set of images, we continued to test the classification using five machine learning models: K-Nearest Neighbor (KNN), Random Forest (RF), Gaussian Naive Bayer (Gaussian NB), Decision Tree (DT) and Support Vector Machine (SVM).

**Figure 8:**
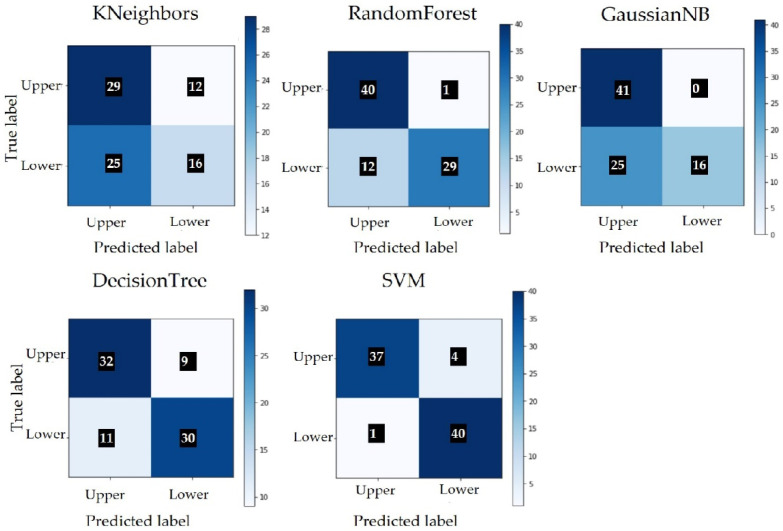
Confusion matrix without normalization.

The extraction of the characteristics was performed starting from the three basic concepts: color, texture and shape. Color features were extracted using histograms; textures were extracted using Haralick features; and shapes were extracted using Hu moments. Several combinations of the three features were tested, including the test of color features based on both color and grey images. The best results were achieved using all three features, color images and the SVM algorithm. The classification results are presented in Table 5 and the confusion matrix is built for each model in Figure 8. Class 0 represents the upper side and class 1 represents the underside of the leaf.

**Figure 9:**
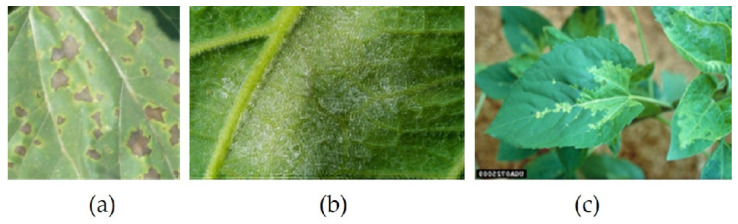
Images incorrectly classified by SVM. Image (**a**) shows the upper side. Image (**b**) shows the lower side. Image (**c**) shows the upper side.

If we compare the classification results of SVM from Table 5 with the classification results of ResNet from Table 4, the number of misclassified images is lower in the case of SVM. Due to the small number of images, we cannot conclude that SVM offers better results than ResNet. More testing and training should be performed with a larger dataset. Figure 9 shows images that were wrongly classified by SVM. Of the five images with a wrong classification, two of them were not included in Figure 9, as they are similar to image Figure 9c [29], modified by rotation.

## 4. Discussion

The description of the diseases presented in Table 1 highlights the different manifestations of foliar diseases on the upper and lower parts of the leaf. Previous studies on the classification of diseases did not consider the analysis of both sides of the leaf as a possible means to improve accuracy. The availability of a set of images remains the main limitation of the research in this field. The used dataset contains only images of sunflower leaves; a test on a diverse set of plants will be conducted in another study.

Two classification methods were considered: one based on deep learning, achieving image classification with the ResNet50 and ResNet152 networks, and for the second, the classification was conducted with five machine learning models.

To identify characteristics relevant for classification, the segmentation of the veins on the leaf was analyzed. The segmentation of the leaf veins with classic edge detection algorithms proved difficult for pictures with a rich context. Therefore, the images obtained by this method could not be used as inputs for implementing a classification model.

Using the CNN model, a high accuracy was achieved on the dataset of images focused on the leaf and veins. Testing the algorithm has highlighted the fact that some images are difficult to classify even for the human eye.

Tests conducted with machine learning algorithms show that elements such as color, shape and texture are relevant in classification. The result obtained using SVM has a higher accuracy than that obtained with CNN.

## 5. Conclusions

Research in the field of classifying plant diseases is strongly limited by the lack of a set of images taken from the field, which contain both background elements and disruptive factors, such as insects, dust, pollen, and sunlight. Although there are many studies related to plant disease detection using artificial intelligence methods, they do not incorporate the diversity of nature. In order to apply the solution of this study on images collected from the field, it is necessary to implement a leaf segmentation system. In recent studies, Mask R-CNN and U-Net have been used to realize leaf segmentation. After segmentation, the solution to classify upper and lower leaf side can be applied. The final step is to classify the disease using the symptoms present on both sides of the leaf. Even in studies in which the classification of the disease is carried out with the help of images of segmented lesions, the use of our solution is necessary.

The analysis of disease symptoms on both sides of a leaf is a topic that has not been addressed in the literature. The first step to introduce this new element into the classification of diseases is represented by the upper–lower leaf side classification. The obtained results can be later used to implement a disease classification algorithm that takes into account the symptoms present on both sides of the leaf. In addition, in a subsequent study, the methods will be applied to a set of images containing leaves of different types of plants.

## Figures and Tables

**Table 1:** Disease symptoms extracted from phytopathology books [23,24].

Disease	Symptoms
Downy Mildew	Primary systemic infection (soil born) is represented by a greenish-yellow color on the upper side and a whitish down on the lower side; Secondary infection occurs in the stage when there are 4 to 8 leaves on the plant. On well-developed leaves, discoloration spots of pale green to yellow or brown areas appear on the upper leaf surface, covered on the underside by a white efflorescence.
White Rust	On the upper leaf surface, there are chlorotic yellowish-green pustules. Two sizes of leaf spots have been observed: large lesions (5–10 mm in diameter), which often coalesce, and smaller lesions (1–2 mm in diameter); On the underleaf, directly opposite, pustules develop to form creamy-white, blister-like structures that appear similar to the pustules of downy mildew.
Alternaria leaf blight	On the upper leaf side, there are circular, dark-brown-to-black lesions with concentric rings ranging from 0.2 mm to 0.5 mm in diameter or circular lesions, with grayish-brown centers, often with accompanying yellow haloes around lesions. Lesions eventually enlarge in size and coalesce, causing the blighting of the leaves. Some lesions can be identified by distinct yellow halos, particularly on young plants.
Septoria leaf spot	The spots begin as water-soaked areas, which are angular with tan centers and brown margins. Narrow yellow haloes often surround young spots. Mature leaf spots may contain tiny black specks, the fungal fruiting bodies (pycnidia). The mature leaf has yellowish-brown lesions, circular or angular, 3–15 mm in diameter, with blackish dots.
Rust	Small pustules develop on both the upper and lower leaf surface and pustule color varies based on the spore stage involved. The upper side of the leaves develops pycnia, which appear on yellow–orange spots (0.6 cm of less). Each spot may be surrounded by a chlorotic halo. Aecia develop on the underside of the leaf, directly opposite the pycnia. Aecia is a collection of small orange-to-yellow cups of the same size as pycnia.

**Table 2:** Dataset used for classification.

Host	Class	No. of Images for Training	No. of Images for Testing
Sunflower	Upper side	252	41
Sunflower	Lower side	252	41

**Table 3:** Training results.

CNN	Image Size	Epochs	Training Loss	Validation Loss	Training Accuracy	Validation Accuracy
ResNet152	224 × 224	6	0.0066	0.09	1	0.9683
ResNet50	224 × 224	6	0.0101	0.0509	1	0.9921

**Table 4:** Classification results obtained on the tested dataset.

ML Model	Image Size	Class	Accuracy	Precision	Recall	F1
ResNet152	224 × 224	0	90%	0.94	0.85	0.89
		1		0.86	0.95	0.90
ResNet50	224 × 224	0	91%	1	0.83	0.90
		1		0.85	1	0.92

**Table 5:** Classification results obtained on the tested dataset.

ML Model	Image Size	Class	Accuracy	Precision	Recall	F1
K-Nearest Neighbor	224 × 224	0	55%	0.54	0.71	0.61
		1		0.57	0.39	0.46
Random Forest	224 × 224	0	84%	0.77	0.98	0.86
		1		0.97	0.71	0.82
Gaussian NB	224 × 224	0	69.5%	0.62	1	0.77
		1		1	0.39	0.56
Decision Tree	224 × 224	0	75%	0.74	0.78	0.76
		1		0.77	0.73	0.75
Support Vector Machine	224 × 224	0	94%	0.97	0.9	0.94
		1		0.91	0.98	0.94
